# Autophagy Regulation on Pyroptosis: Mechanism and Medical Implication in Sepsis

**DOI:** 10.1155/2021/9925059

**Published:** 2021-06-24

**Authors:** Ran Guo, Hao Wang, Na Cui

**Affiliations:** Department of Critical Care Medicine, State Key Laboratory of Complex Severe and Rare Diseases, Peking Union Medical College Hospital, Chinese Academy of Medical Science and Peking Union Medical College, Beijing 100730, China

## Abstract

Sepsis is defined as a life-threatening disease involving multiple organ dysfunction caused by dysregulated host responses to infection. To date, sepsis remains a dominant cause of death among critically ill patients. Pyroptosis is a unique form of programmed cell death mediated by the gasdermin family of proteins and causes lytic cell death and release of proinflammatory cytokines. Although there might be some positive aspects to pyroptosis, it is regarded as harmful during sepsis and needs to be restricted. Autophagy was originally characterized as a homeostasis-maintaining mechanism in living cells. In the past decade, its function in negatively modulating pyroptosis and inflammation during sepsis has attracted increased attention. Here, we present a comprehensive review of the regulatory effect of autophagy on pyroptosis during sepsis, including the latest advances in our understanding of the mechanism and signaling pathways involved, as well as the potential therapeutic application in sepsis.

## 1. Introduction

Sepsis refers to a dysregulated host response against pathogens and the ensuing life-threatening multiorgan dysfunction [1]. Sepsis is an important public health issue that is associated with high morbidity in many intensive care units. At present, the treatment of sepsis is still dominated by fluid resuscitation and the use of antibiotics, although these nonspecific therapies cannot reduce the high mortality of sepsis. Research focusing on the pathogenesis of sepsis is needed, especially for the development of new therapies.

**Figure 1:**
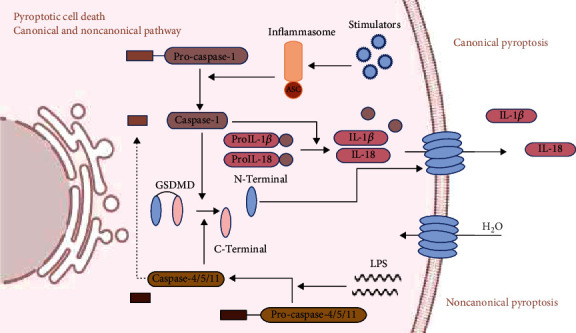
Canonical and noncanonical pyroptosis pathways. The canonical pyroptosis pathway requires the involvement of inflammasomes. Inflammasomes are multiprotein complexes assembled in response to external stimuli, such as hypoxia, injury, toxins, and pathogens. A typical inflammasome contains a pattern recognition receptor and a downstream adaptor such as apoptosis-associated speck-like (ASC) protein. Four main inflammasomes have been identified: NLR members NOD-, LRR-, and pyrin domain containing 1 (NLRP1); pyrin domain containing 3 (NLRP3); NOD-, LRR-, and caspase recruitment domain containing 4 (NLRC4); and absent in melanoma 2 (AIM2), a sensor for nucleic acids. Different inflammasomes receive different stimulatory signals. Activation of inflammasomes promotes maturation of procaspase-1 into caspase-1, which further cleaves immature prointerleukin- (pro-IL-) 1*β* and pro-IL-18 into mature IL-1*β* and IL-18. Caspase-1 cleaves GSDMD into N- and C-terminal components. The N-terminal domains bind to cell membranes to form oligomeric pores, causing lytic cell death. In the noncanonical pyroptosis pathway, caspase-11 and caspase-4/5 are involved and inflammasomes are not needed. The stimulatory signals mainly come from Gram-negative bacterial lipopolysaccharide (LPS). LPS activates caspase-11 (caspase-4/5 in humans) directly and mature caspase-4/5/11 cleave GSDMD to trigger pyroptosis. Caspase-4/5/11 do not have the function to process pro-IL-1*β* and pro-IL-18 into mature IL-1*β* and IL-18. However, inflammatory cytokines released in the noncanonical pyroptosis pathway have been observed, which indicates the probable interaction between the two pathways.

Pyroptosis is a unique form of programmed cell death mediated by the gasdermin (GSDM) family of proteins. This process occurs when cells receive stimulatory signals, including pathogen-associated molecular patterns (PAMPs) and damage-associated molecular patterns (DAMPs), and results in lytic cell death and release of proinflammatory cytokines interleukin- (IL-) 1*β* and IL-18 (Figure 1) [2–4]. In recent years, cell pyroptosis has proved to play an important part in the progression of many diseases including cancer and autoimmune, cardiovascular, and infectious diseases [5–8], as well as sepsis [9, 10]. In general, pyroptosis occurs when host organisms are infected by pathogens, resulting in cell membrane rupture and abundant release of proinflammatory cytokines, thus blocking intracellular bacterial replication and recruiting immune cells to eliminate the pathogens. Pyroptosis has a protective effect to some degree [11, 12]; however, it is also responsible for sepsis and septic shock when overactivated [9]. Pyroptosis of neutrophils and monocytes is a major source of IL-18 during infection [13]. An increased serum IL-18 level indicates the severity of sepsis and is correlated with worse prognosis [14, 15]. A high concentration of serum IL-1*β* also has an adverse effect on the prognosis of septic shock [16, 17]. Organ damage caused by overactivated pyroptosis has been demonstrated in many experiments. It is important to eliminate the adverse effect of pyroptosis in sepsis.

Autophagy is an important mechanism for maintaining homeostasis in the intracellular environment and has a regulatory effect on pyroptosis. Autophagy refers to the process of selective degradation of macromolecules and damaged organelles through the lysosomes, and it is one of the most important homeostasis-maintaining mechanisms in living cells. During sepsis, autophagy regulates differentiation, renewal, and energy metabolism of various cell types, including histiocytes and immune cells [18]. In sepsis, autophagy is a protective mechanism against multiple organ dysfunction [19]. Autophagy has modulatory effect on pyroptosis. A study in 2008 suggested that the loss of autophagy-related proteins (ATGs) enhanced the release of IL-1*β* and cell lysis following pyroptosis. This was when researchers began to focus on the interplay between these two cellular events [20]. In the present review, we introduce the latest research findings about the regulatory effect of autophagy in pyroptosis, including the underlying mechanism as well as its potential application in the treatment of sepsis.

## 2. What Do We Know about Pyroptosis in Sepsis

Pyroptosis is an ideal tool for the clearance of infected cells because it is a form of lytic cell death that causes release of abundant inflammatory cytokines and enables destruction of intracellular replicative niches. This might be the positive role of pyroptosis in infection, but if out of control, it could be detrimental to the host [21–24]. Pyroptosis contributes to the hyperinflammatory response in sepsis, and recent advances indicate its role in adaptive immunity, which is closely related to immune paralysis. Pyroptosis is a double-edged sword in sepsis, but the negative effect plays the dominant role.

Current theories regard pyroptosis as a part of innate immunity. Pyroptotic cells release IL-1*β* and IL-18 and then stimulate and recruit neutrophils and macrophages to the focus of infection [25], which may be an effect of pyroptosis in the early stage of sepsis. However, there are some research findings that deemphasize this function of pyroptosis in innate immunity. The NOD-like receptor family, pyrin domain containing 3 (NLRP3), is a well-explored inflammasome, and its activation can be an important initiating factor in pyroptosis. In NLRP3-deleted mice, total white blood cell and neutrophil recruitment to the peritoneal cavity were similar to that in wild-type mice at 6 and 24 h following cecal ligation and puncture (CLP), and neutrophils showed augmented phagocytotic ability. We can almost conclude that NLRP3 activation does not have a positive effect on neutrophil recruitment and bacterial clearance during sepsis [26]. Recently, nonpyroptotic secretion of IL-1 has attracted scientists' attention [27]. This mechanism has been discovered in macrophages and neutrophils. A hypothesis has been put forward that macrophages undergo a hyperactivated state between activated and pyroptotic states. In the state of hyperactivation, macrophages release IL-1*β* in a GSDMD-dependent manner, dispensing with pyroptotic death. This mechanism minimizes the cost of inflammatory response and maximizes the benefit, preserving the number of effective macrophages. GSDMD-mediated nonpyroptotic IL-1 release has also been observed in neutrophils [13], and cell destiny may be influenced by different stimuli. This mechanism prevents neutrophils from excessive consumption and maintains a persistent reaction.

Research has uncovered the interaction between pyroptosis and adaptive immunity. One widely accepted belief is that pyroptosis serves as a modulator of adaptive immunity. IL-18 released from pyroptotic cells can stimulate T cells and natural killer cells to produce interferon- (IFN-) *γ* [25]. IL-1 and IL-18 also regulate CD4^+^ T cells for cytokine transcription [28]. Additionally, IL-1 can stimulate production of IL-6 and then regulate B-cell proliferation and antibody production [25].

Adaptive immune cells are not just targets of pyroptotic products. They undergo pyroptosis themselves. Pyroptosis exists in adaptive immune cells such as T lymphocytes and B lymphocytes, causing a sharp decrease in the number of these cells, leading to immunosuppression. In autoimmune diseases such as rheumatoid arthritis, lymphocytes have been confirmed to undergo NLRP3-mediated pyroptosis [29]. In HIV infection, caspase-3-mediated apoptosis has been proposed as the key mechanism for CD4^+^ T cell loss. However, we now know that most CD4^+^ T cells die because of caspase-1-mediated pyroptosis. Additionally, dying CD4^+^ T cells release DAMPs that induce more uninfected cells to die, creating an inflammatory cycle and leading to immune deficiency [30]. Even if adequate antiviral drugs are used and viral replication is suppressed, persistent rounds of pyroptosis still exist. This virus-independent manner of CD4^+^ T cell death contributes to the long-term immune impairment [31]. In a lipopolysaccharide (LPS)/ATP-induced murine splenic lymphocyte pyroptosis model, scientists observed accelerated transcription of the NLRP3 inflammasome and upregulated caspase-1 activity, as well as increased release of cytokines and pyroptosis [32]. There is an immunosuppressed stage in sepsis, and this results from the loss of conventional (e.g., CD4^+^ and CD8^+^) T cells and Th17 cells and upregulation of regulatory T cells [33]. Whether pyroptosis leads to a sharp decrease in lymphocyte number in sepsis and causes diminished immune function deserves further investigation. To date, there have been few studies to support the presence of lymphocyte pyroptosis in sepsis, either in animal models or in clinical specimens. It deserves further investigation whether lymphocytes undergo pyroptosis during sepsis and whether we can attribute the immune paralysis to lymphocyte pyroptosis.

## 3. Autophagy Is a Negative Modulator of Pyroptosis

### 3.1. Two Widely Accepted Molecular Mechanisms

Autophagy can negatively regulate pyroptosis. This viewpoint has been put forward based on a series of findings about the correlation between ATGs and pyroptosis. ATGs are indispensable in the execution of autophagy, including phagophore initiation, expansion, transition, and fusion with lysosomes [34]. In 2008, Saitoh and colleagues revealed that the loss of Atg16L1 led to overactivation of the canonical pyroptosis pathway, together with the abundant release of inflammatory cytokines [20]. Subsequent research revealed the relationship between Atg7 and pyroptosis. In a mouse model of *Pseudomonas aeruginosa*-induced sepsis, Atg7 gene knockout mice showed enhanced activity of inflammasomes in macrophages, accompanied by elevated blood levels of IL-1*β* and IL-18 and increased pyroptosis in macrophages. All these changes led to a deficiency in pathogen clearance and exacerbated inflammatory lung injury, which shortened the survival time of the animals [35]. In recent years, scientists have recognized a family of immunity-related GTPase M clade (IRGM), which is related to autophagy through direct interaction with members of the Atg8 family, and control of membrane fusion events. It has been suggested that IRGM proteins modulate LPS-induced caspase-11 activation and noncanonical pyroptosis in sepsis models both *in vivo* and *in vitro*, perhaps through modulating autophagy [36, 37]. These findings demonstrate that autophagy is a key player in the regulation of cell pyroptosis. In the past decade, research has revealed various mechanisms underlying this regulation, which can be summarized as follows. Autophagy blocks pyroptosis through elimination of DAMPs and PAMPs and directly targeting the essential components involved in this process.

#### 3.1.1. Autophagic Elimination of DAMPs and PAMPs

Pyroptosis can be divided into canonical and noncanonical pathways, according to the different initiating factors and effective molecules. The canonical pyroptosis pathway requires the involvement of inflammasomes (Figure 1). Inflammasomes are protein complexes assembled in response to a variety of stimuli, including PAMPs and DAMPs [38]. DAMPs are defined as endogenous molecules that can initiate and potentiate inflammatory responses, while PAMPs refer to exogeneous microbial products, such as LPS [39, 40]. They are both recognized by PRRs, which are an element of inflammasomes. LPS can directly activate caspase-11-mediated noncanonical pyroptosis, without participation of inflammasomes or PRRs. Autophagy downregulates pyroptosis through eliminating DAMPs and PAMPs.

Numerous molecules serve as DAMPs that initiate and potentiate noninfectious inflammatory responses during sepsis [i.e., high mobility group box 1 (HMGB1), histones, ATP, uric acid, and interleukin] [39]. In addition to these, a group of DAMPs, including reactive oxygen species (ROS), mitochondrial DNA (mtDNA), and reactive nitrogen species (RNS), have gained special attention because they are intimately related to mitochondrial damage. Mitochondria are the major site of ROS generation, and ROS are activators of the NLRP3 inflammasome, which is widely recognized as a promoter of pyroptosis [41, 42]. Besides ROS, mtDNA also has the potential to promote pyroptosis. When mitochondria are damaged or dysfunctional, ROS and mtDNA are released into the cytoplasm, leading to overactivation of the NLRP3 inflammasome. The selective autophagic degradation of mitochondria is defined as mitophagy, and it plays an important role in maintaining mitochondrial integrity and homeostasis in living cells, thus inhibiting cellular inflammatory response and pyroptosis. In cells with mitophagic deficiency, there are exacerbated mitochondrial dysfunction and paramorphia, including mitochondrial membrane permeability transition, through which ROS are released into the cytoplasm, leading to overactivation of NLRP3 and caspase-1 [43]. Increased ROS generation is also a promoter of autophagy, which occurs prior to inflammasome activation. In the early phase of *Streptococcus pneumoniae* infection, ROS can stimulate cell autophagy and in turn inhibit pyroptosis, thus preventing cell death [44]. In Leydig cells, negative modulation of pyroptosis by autophagy occurs through elimination of overproduced ROS and damaged mitochondria [45]. Apart from ROS, the mitochondria are a major source of RNS in living cells [46]. Research has revealed that RNS promote pyroptosis [47]. It is also probable that autophagy inhibits pyroptosis through clearance of mitochondrial RNS.

The noncanonical pyroptotic signaling pathway is also under the regulation of autophagy. It is widely recognized that LPS is the major activator of caspase-11-mediated noncanonical pyroptosis, which renders the host sensitive to sepsis and septic shock [4, 48]. Autophagosomes take up and degrade vacuolar Gram-negative bacteria and reduce the activation of caspase-11-mediated noncanonical pyroptosis. In 2014, Meunier and colleagues conducted a study in a model of *Salmonella typhimurium*-infected macrophages and proved the inhibitory effect of autophagy on noncanonical pyroptosis through eliminating PAMPs [49]. A similar hypothesis was put forward by Roberts and colleagues in 2015. In Gram-negative bacterial infection, caspase-11- (4/5 in humans) mediated pyroptosis was prevented by enhanced autophagic clearance of pathogens [50]. Suppression of noncanonical pyroptosis may help to resist LPS-induced death.

#### 3.1.2. Autophagic Clearance of Indispensable Components in Pyroptosis

Autophagy can also exert its inhibitory effect on pyroptosis through directly targeting the essential components involved in pyroptosis, such as inflammasomes and their downstream molecules. It is reported that a variety of inflammasomes [i.e. absent in melanoma 2 (AIM2), NLRP1, and NLRP3] as well as inflammasome component ASC (apoptosis-associated speck-like protein containing a caspase recruitment domain) can be taken up and degraded via autophagy after ubiquitin modification [38, 51–53]. In 2011, Harris and colleagues found that, in bone-marrow-derived macrophages and dendritic cells, pro-IL-1*β*, the precursor of cytokine IL-1*β*, could be directly degraded by autophagy, resulting in reduced release of IL-1*β*. This effect could be reinforced by mammalian target of rapamycin (mTOR) antagonist rapamycin [54].

Pyroptosis is GSDM-mediated programmed cell death. The GSDM family of proteins has many members. Humans harbor GSDMA, GSDMB, GSDMC, GSDMD, DFNA5, and DFNB59. They all contain an N-terminal domain that can promote mammalian cell pyroptosis [2]. GSDMD is the most widely studied. When cleaved by caspase-1 or caspase-4/5/11, the N-terminal domain of GSDMD is released and forms cytotoxic pores in the cell membrane [9]. Researchers have found that autophagy prevents pyroptosis through downregulation of cleaved GSDMD level [55]. A recent study reported that rapamycin, an autophagy agonist, reversed GSDMD-mediated pyroptosis after LPS stimulation. The authors also found that rapamycin alone did not exert an inhibitory effect on pyroptosis and emphasized the important role of autophagy in the entire process [56].

Autophagy also plays a modulatory role in GSDME-mediated pyroptosis, while the mechanism is still unclear. GSDME can be cleaved by activated caspase-3 to form the cytotoxic N-terminal fragment in mammals. In teleost fish, GSDME can be cleaved by caspase-1 and caspase-7 and oligomerize to form GSDME pores in the plasma membrane. The expression level of GSDME determines cell fate: cells containing sufficient GSDME undergo pyroptosis, and cells that lack GSDME undergo apoptosis [57]. In a study of human melanoma cells in which autophagy was inhibited by chloroquine, enhanced pyroptosis was mediated by GSDME. This research did not reveal the mechanism underlying the interplay between autophagy and GSDME-mediated pyroptosis, whereas the AMPK-eEF-2K signaling pathway is implied to be of importance in that process [58].

### 3.2. Autophagy, Pyroptosis, and Some Classic Signaling Pathways

**Figure 2:**
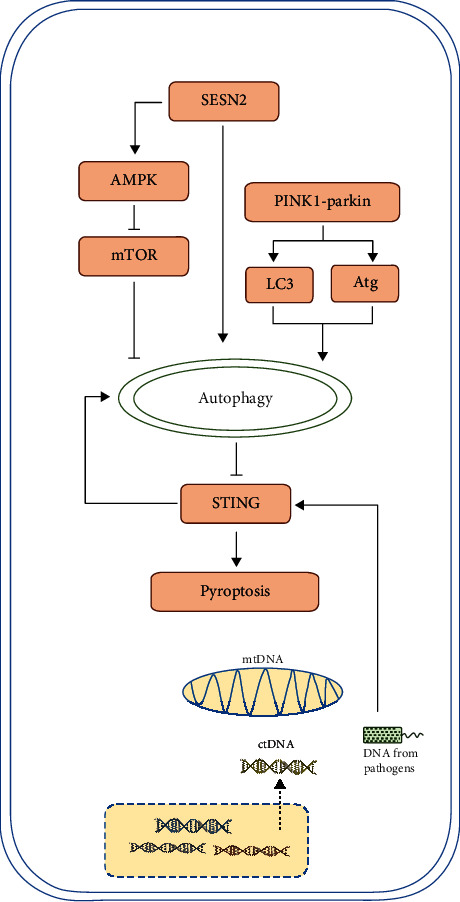
Autophagy, pyroptosis, and some classic signaling pathway. (1) Autophagy is under control of mTOR signaling and PINK1-parkin signaling. SESN2 signaling pathway upregulates autophagy through inhibiting mTOR or other mechanisms. Research indicate these signaling pathways have further impact on pyroptosis level. (2) STING senses DNA derived from damaged organelles or pathogens and get activated, which induces both pyroptosis and autophagy. Autophagy in turn negatively modulates STING activity and restricts overactivated pyroptosis.

There are many popular pathways which may be involved in the modulation of autophagy on pyroptosis, but their exact role has not been elucidated yet (Figure 2). Signals such as PINK1–parkin, SESN2, and mTOR are upstream of autophagy, and signals such as STING are downstream of autophagy but upstream of pyroptosis. Recent studies showed some interesting interaction.

Inhibition of NLRP3 has a protective role in lethal sepsis through neuroimmune modulation [59]. The PINK1-parkin pathway is important in this process. PINK1 is a protein kinase that monitors mitochondrial integrity, and Parkin is an ubiquitin–protein ligase that serves as a degradation signal of mitochondria. They cooperatively facilitate autophagic clearance of damaged mitochondria, and their role in neurological disorders has been well studied. In PINK1–PARK2 gene knockout mice, a decrease in the circulating level of the neurotransmitter dopamine has been observed, as well as activation of NLRP3 inflammasome and overexpression of downstream sepsis mediator, HMGB1. It is recognized that the PINK1–parkin pathway triggers mitophagy and plays a major role in maintaining mitochondrial homeostasis during sepsis [60, 61]. In another study, scientists found that the mitophagy/autophagy system acted as a suppressor of NLRP3 inflammasome activation through scavenging mitochondrial ROS [62]. Whether there is a connection between the PINK1–PARK2 pathway and these modulatory mechanisms remains to be further investigated.

Stimulator of interferon genes (STING) is related to uncontrolled pyroptosis and is an important factor in sepsis [63]. Cytosolic DNA serves as an activator of STING through GMP-cGAMP pathway and triggers pyroptosis through both canonical and noncanonical pathways [64]. It has been shown that the cytosolic DNA–STING–pyroptosis axis is under autophagic regulation [65, 66]. Key genes involved in autophagy, such as ULK-1 and Atg9, have been shown to suppress STING signal and inhibit inflammation and pyroptosis. Interestingly, autophagy can be intrinsically induced by STING trafficking [67]. The balance among autophagy, STING signaling, and pyroptosis needs further investigation.

There is crosstalk between parkin or PINK1 and the STING pathway. In mice lacking parkin or PINK1, STING-mediated type I IFN response is overactivated. Parkin or PINK1 has the potential to clear damaged mitochondria through induction of mitophagy, thus preventing mtDNA release and STING activation [68]. These findings attach importance to modulation of autophagy to restrict inflammation and could offer a novel therapeutic strategy in sepsis.

SESN2, a stress-inducible protein, suppresses prolonged NLRP3 inflammasome activation by inducing mitophagy and clearing damaged mitochondria in macrophages. In macrophages, after stimulation by LPS, increased NO synthase 2 generates NO and then upregulates the level of SESN2. SESN2 induces mitophagy by inducing aggregation of sequestosome-1 and its binding to Lys63-ubiquitinated mitochondria and maintaining the ULK1 protein level [69].

Both autophagy and pyroptosis were under the regulation of a peroxisome proliferator-activated receptor- (PPAR-) *γ*-dependent pathway. When LPS-challenged macrophages were treated with a PPAR-*γ* pathway activator, enhanced apoptosis and inhibited autophagy were observed. A decrease in the expression of NLRP3 and a reduction in IL-1*β* levels were also found. Although the direct regulation between autophagy and pyroptosis was not investigated in this study, we can hypothesize that there may be a close correlation between them [70].

Signaling pathways that regulate autophagy have been confirmed to regulate pyroptosis. Among these, the mTOR signaling pathway has attracted much attention. mTOR controls cell growth and metabolism [71, 72]. In a variety of cells, mTOR regulates programmed cell death through modulating autophagy level. mTOR regulation of apoptosis has been investigated systematically [19, 73], whereas there have been fewer studies on pyroptosis, but they are increasing gradually. The mTOR signaling pathway can be blocked by different drugs, which upregulates the autophagy level and inhibits pyroptosis. One research team revealed that blockade of the mTOR signaling pathway influenced cell autophagy and pyroptosis. They used rapamycin, a specific antagonist of mTOR, to block mTOR signaling in a cecal legation and puncture- (CLP-) induced sepsis mouse model. Rapamycin inhibited pyroptosis of macrophages and reduced the release of inflammatory cytokines. This phenomenon was associated with activated autophagy. In the rapamycin-treated mice, inflammatory impairment in the lungs, liver, and spleen was attenuated [74]. Li and colleagues found that adrenomedullin had protective effects on pyroptosis and biological functions of Leydig cells exposed to LPS, by promoting autophagy. That effect was closely related to inhibition of the ROS–AMPK–mTOR signaling pathway and the clearance of intracellular ROS and damaged mitochondria [45].

## 4. More Complicated Interplay between Autophagy and Pyroptosis

As previously discussed, autophagy can negatively regulate pyroptosis and inflammatory cytokine release through elimination of DAMPs or PAMPs, inflammasomes, and other essential pyroptotic components. This theory is supported by a mass of research findings. Whether autophagy acts as a negative modulator of pyroptosis under any circumstances is full of controversy. Some studies have drawn the opposite conclusion that autophagy not only fails to inhibit pyroptosis but also plays a promotive role.

In a study of bone marrow-derived macrophages, starvation enhanced autophagy while the pyroptosis level was not reduced accordingly [75]. In contrast, the activity of inflammasomes and release of IL-18 and IL-1*β* were upregulated. Yuan and colleagues constructed a model of benzo[a]pyrene-induced hepatic cell pyroptosis. When treated with 3-methyladenine, a well-known autophagy inhibitor, the levels of autophagic flux and pyroptosis-related proteins (i.e., procaspase-1, activated caspase-1, cyclo-oxygenase-2, IL-1*β*, and IL-18) were reduced, indicating impaired pyroptosis [76]. It is believed that there is a mutual reinforcement between pyroptosis and autophagy. In another study, scientists detected that starvation-induced autophagy enhanced extracellular release of IL-1*β*, which is opposite to the effect of basal autophagy [75]. A hypothesis has been put forward in which production of ATP, a second signal to stimulate inflammasome activation, is under the control of autophagy. When autophagy is inhibited, ATP production is also reduced, which dampens the downstream inflammasome assembly and cytokine release [54, 77, 78].

It is unclear why there is a discrepancy in the results of the above studies. One possible reason is that the different pathological conditions and cell types may have influenced the direction of autophagic modulation towards pyroptosis. Besides, *in vitro* experiments have some intrinsic limitations in simulating real pathological conditions. The heterogeneity of different experiments is also a possible reason. The regulatory effect of autophagy on pyroptosis requires further investigation, and there may be some unknown mechanisms that contribute to the complicated regulation. In future investigations, the time when autophagy inhibits the activation of inflammasomes and reduces pyroptosis must be established, so that we can use autophagy induction as a therapeutic approach in patients with sepsis.

Another view is that autophagy and pyroptosis have synergistic effects and they are both used by cells to eliminate pathogens and limit infection [79]. During a physiologic condition without any stimulator, autophagy components Beclin-1/Atg6 are bound with NLRC4, and autophagy is inhibited. When contaminated by bacterial component flagellin, NAIP5 binds NLRC4 and Beclin-1/Atg6 are released to initiate autophagy. When the contaminants exceed the capacity of autophagic elimination, NLRC4 cleaves caspase-1 and sufficient caspase-1 triggers pyroptosis, initiating an inflammatory response that eliminates the pathogen's protected niche.

Autophagy limits inflammasome activity by direct engulfment, and activation of inflammasomes can in turn stimulate the formation of autophagosomes [53]. This is regarded as a mechanism that protects cells from pyroptotic death [79]. This has been supported by another study in NLRP3-deficient mice subjected to CLP, in which peritoneal cells showed decreased autophagy [26]. In many diseases characterized by excessive inflammation, such as sepsis, inflammasomes are not adequately cleared by autophagy. The inflammatory signals then trigger pyroptosis, leading to exaggerated inflammation and organ damage. Why this negative-feedback loop fails to eliminate inflammasomes and how to make this modulatory effect more effective need further investigation.

## 5. A New Target in Sepsis Treatment

**Figure 3:**
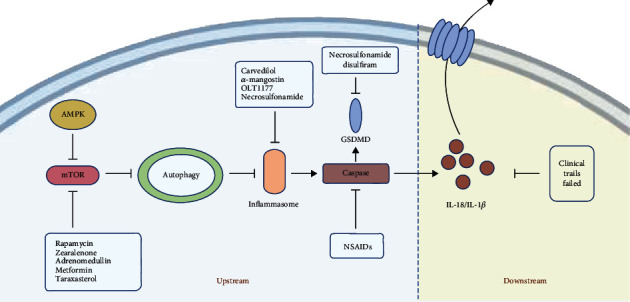
Autophagy-pyroptosis axis and potential therapeutic targets. New drugs have been developed to target every component in this axis and can be divided into upstream and downstream molecules. Prior research targeting the downstream molecules such as interleukin- (IL-) 18 and IL-1*β* failed. Recent advances have been made in the development of drugs targeting the upstream molecules, including inflammasomes, caspases, and GSDMD. NSAIDs: nonsteroidal anti-inflammatory drugs.

There is currently a lack of effective treatment for sepsis. An improved understanding of the mechanisms underlying sepsis has led to novel therapeutic strategies. As discussed above, exaggerated pyroptosis does more harm than good in sepsis. Therefore, scientists have begun to explore pyroptosis-inhibiting therapies for sepsis. Trials assessing the therapeutic benefit of blocking inflammasomes and their downstream components have been carried out. Some studies have focused on the final products of pyroptosis, such as IL-18 and IL-1*β*, and have ended in failure, which seems to confirm that the inhibition of downstream cytokines is not feasible. IL-1 blockade impairs innate immune response and increases the risk of opportunistic infections [80]. Perhaps, we should turn our attention to the upstream signals that cause the release of these cytokines [13, 81]. New drugs have been developed that target inflammasomes, caspase, and GSDM proteins, and there has been a new understanding of traditional medicines, and all the findings possess some translational value in various conditions including autoimmune diseases, metabolic diseases, neurodegenerative diseases, and cancers [80]. Necrosulfonamide, which is reported to be a GSDMD inhibitor, also inhibits NLRP3 to prevent pyroptosis. Disulfiram is an FDA-approved drug for treating alcohol addiction, and a recent study found it was an inhibitor of pore formation by GSDMD in pyroptosis (Figure 3) [82–87]. Autophagy has been shown to have a regulatory effect on pyroptosis, enabling us to cure sepsis through inducing autophagy and inhibiting pyroptosis.

In 2019, Ge and colleagues showed that *α*-mangostin-induced autophagy inhibited LPS-stimulated NLRP3 inflammasome activation in macrophages and then reduced IL-1*β* release. Phagocytosis of macrophages was enhanced, and organ function was restored [88]. All that was missing was discussion about the mechanism underlying the regulatory effect of *α*-mangostin on autophagy and pyroptosis. Taraxasterol, an extract from another Chinese traditional medicine dandelion, suppresses the activation of caspase-1 in LPS-treated murine macrophages. Taraxasterol displays its anti-inflammatory effects likely through regulation of the mTOR signaling pathway [89].

Metformin, a classic antidiabetic drug, has been shown to have a protective effect in patients with sepsis. Metformin use prior to hospital admission has been associated with a significantly lower mortality rate in patients with sepsis [90]. In many pathological conditions such as ischemia–reperfusion injury [90], inflammatory bowel disease [91], and diabetic cardiomyopathy [92], metformin ameliorates pyroptosis. One probable mechanism of this protective effect is that metformin inhibits inflammasome NLRP3 through enhancing autophagy [91], and the AMPK–AKT–mTOR signaling pathway is critical in that process [92].

The *α*/*β*-blocker carvedilol (CVL) is traditionally used for cardiovascular disorders. Recent studies have revealed the anti-inflammatory effect of CVL. CVL induces autophagy, inhibits activation of NLRP3, and reduces pyroptosis. CVL may cause autophagic induction through a sirtuin-1-dependent pathway [93]. Some animal experiments have suggested a protective effect of low-dose CVL on septic kidney injury. CVL has ameliorated renal perfusion pressure in rats and reduced serum levels of blood urea nitrogen and creatinine. This renal protective function of CVL may be related to its anti-inflammatory effect [94].

Pharmaceutical treatment for sepsis through inducing autophagy and inhibiting pyroptosis remains in the preclinical stage. Current research is not adequate to provide evidence whether these drugs are beneficial for prognosis and survival rate, nor does it reveal potential adverse effects and long-term immunodeficiency. Autophagy has a complicated two-sided modulatory effect on pyroptosis, which is strongly linked with cell type and pathological condition. Therefore, more basic research is needed to explore the modulatory mechanism of autophagy on pyroptosis and to establish proper therapeutic targets. If the inhibition of pyroptosis can be achieved in specific cells without influencing other cells, there could be an exciting breakthrough in the treatment of sepsis.

## 6. Conclusion and Perspective

Pyroptosis is a relatively new research area, and the mechanism underlying the modulatory effect of autophagy on pyroptosis has not been clarified. Pyroptosis is closely related to the pathophysiological process of sepsis. Autophagy can negatively modulate pyroptosis and minimize the harmful effect of pyroptosis, which may be a new target in sepsis treatment in the future. At present, there are limitations to basic research in this area, and drug development remains in the early stage. An in-depth understanding of autophagic regulation of pyroptosis is expected to bring a new dawn for sepsis treatment.

## Abbreviations

PAMPs: Pathogen-associated molecular patterns
DAMPs: Damage-associated molecular patterns
IL-1*β*: Interleukin-1*β*
IL-18: Interleukin-18
ATGs: Autophagy-related genes
ROS: Reactive oxygen species
NLRP3 inflammasome: NOD-like receptor family, pyrin domain containing 3 inflammasome
mtDNA: Mitochondrial DNA
RONS: Reactive oxygen and nitrogen species
ASC: Apoptosis-associated speck-like protein containing a caspase recruitment domain
mTOR: Mammalian target of rapamycin
GSDME: Gasdermin E
3-MA: 3-Methyladenine
CVL: Carvedilol.
